# Diabetes Mellitus after SARS-CoV-2 Infection: An Epidemiological Review

**DOI:** 10.3390/life13061233

**Published:** 2023-05-23

**Authors:** Irena Ilic, Milena Ilic

**Affiliations:** 1 Faculty of Medicine, University of Belgrade, 11000 Belgrade, Serbia; 2 Department of Epidemiology, Faculty of Medical Sciences, University of Kragujevac, 34000 Kragujevac, Serbia; drmilenailic@yahoo.com

**Keywords:** SARS-CoV-2, diabetes mellitus, epidemiology, risk factors

## Abstract

Diabetes mellitus (DM) is among the major global public health issues. According to recent projections, a continued rise in DM prevalence is expected in the following decades. The research has shown that DM is associated with poorer outcomes of coronavirus disease 2019 (COVID-19). However, there is growing evidence suggesting that COVID-19 is associated with new-onset DM type 1 and type 2. This review aims to summarize the current knowledge about the new onset of DM following COVID-19. All identified studies were longitudinal, and they have predominantly shown a significantly increased risk for new-onset DM (both type 1 and type 2) following a SARS-CoV-2 infection. Increased risk of poorer COVID-19 outcomes (mechanical ventilation, death) was noted in persons with new-onset DM following SARS-CoV-2 infection. Studies investigating risk factors for new-onset DM in COVID-19 patients showed that severe disease, age, ethnicity, ventilation, and smoking habits were associated with DM occurrence. The information summarized in this review presents a valuable source of evidence for healthcare policymakers and healthcare workers in the effort of planning prevention measures for new-onset DM after SARS-CoV-2 infection and the timely identification and appropriate treatment of patients with COVID-19 who could be at greater risk for new-onset DM.

## 1. Introduction

Globally, based on a report by the World Health Organization on 27 January 2023, there have been over 752 million confirmed cases of coronavirus disease 2019 (COVID-19), including over 6.8 million deaths [1]. Following the onset of the COVID-19 pandemic, studies that came after have shown that diabetes is among the major risk factors for severe disease, and there has been a rise of all-cause deaths and COVID-19-related deaths among patients with diabetes [2,3,4,5,6,7,8]. A matched population-based case–control study in Scotland showed that rate ratios for severe COVID-19 were 2.75 for type 1 diabetes and 1.60 for type 2 diabetes [9].

In 2021, the International Diabetes Federation estimated that 537 million adults were living with diabetes mellitus (DM) worldwide and predicted a continued rise in global prevalence to 643 million cases by 2030, with forecasts to reach 783 million cases by 2045 [10,11]. Additionally, it is estimated that about 1.2 million children and adolescents (0–19 years) are living with diabetes type 1 worldwide in 2021. Diabetes type 1 (T1DM, which accounts for 5–10% of all diabetes cases) is characterized by a lack of insulin due to autoimmune destruction of the β-cells of the pancreas due to an unknown cause, while diabetes type 2 (T2DM, which accounts for 90–95% of all diabetes cases) is characterized by insulin resistance and relative (rather than absolute) insulin deficiency [12].

Diabetes mellitus is a multi-factorial disease determined by the interaction of genetic factors and environmental exposures [13,14,15]. Viral infection was identified as one of the environmental risk factors that was suspected of playing an etiologic/triggering role in diabetes mellitus occurrence. The most common viruses associated with diabetes mellitus were coxsackieviruses B1–B6, measles, mumps, rubella, varicella, and influenza [13,15]. Notably, people with diabetes are prone to infections, both community-acquired and hospital-acquired (pneumonia, skin and soft tissue infections, sepsis, etc.) [13]. A recent meta-analysis including data from 6,653,207 SARS-CoV-2 patients in Europe showed that an elevated risk for adverse outcomes from COVID-19 (mortality, intensive care unit, and hospital admission) was associated with a history of diabetes [16].

With the growing COVID-19 pandemic, the world is facing a rise in newly diagnosed diabetes cases after infection with COVID-19 [17,18]. A recent meta-analysis showed that patients with severe COVID-19 were nearly twice as likely to contract diabetes after COVID-19 compared to non-COVID-19 patients, particularly in the first 3 months after COVID-19 [19]. Some studies have suggested the association of the incidence of new-onset DM with the severity of COVID-19, positive family history of DM, severity at admission, oxygen duration, higher body mass index, steroid dosage and duration of use, and comorbidity, but findings were not consistent [20,21]. This review aims to summarize the current knowledge about the new onset of diabetes mellitus following COVID-19.

## 2. Materials and Methods

### 2.1. Study Design

A narrative review of the literature was performed. The methodological approach for a nonsystematic literature review proposed by Grant et al. [22] was applied. The research question pertained to the epidemiology of an incident of diabetes following SARS-CoV-2 infection; therefore, the occurrence of new-onset DM in COVID-19 patients compared to persons without COVID-19 was researched.

### 2.2. Data Source

The following databases were searched: PubMed, Scopus, Web of Science, and Google Scholar. For the search terms, combinations of these keywords were used: “diabetes mellitus”, “incidence”, “incident”, “new onset”, “newly diagnosed”, “COVID-19”, and “SARS-CoV-2”. There were no language restrictions. Searches were conducted up to January 2023.

### 2.3. Inclusion and Exclusion Criteria

Studies that matched our inclusion criteria and did not meet our exclusion criteria were considered.

The inclusion criteria comprised cohort and case–control studies that evaluated the burden and risk of new-onset diabetes mellitus following a SARS-CoV-2 infection, which provided data for the diagnosis of SARS-CoV-2 infection, and clearly reported the new diagnosis of diabetes in persons with no previous history of diabetes.

Exclusion criteria were studies that assessed the burden of new-onset diabetes mellitus during the pandemic without focusing on or providing separate data on whether the newly diagnosed diabetes occurred in individuals after a SARS-CoV-2 infection, studies that evaluated the outcomes of COVID-19 in persons who already had diabetes prior to SARS-CoV-2 infection, animal and experimental studies, reviews, meta-analyses, case reports, and case series. In cases when there were several publications on the same population, we used the most recent report and that with the most data. Additionally, reference lists of included studies and meta-analyses, and reviews were hand-searched for any relevant studies.

### 2.4. Ethical Considerations

The study was approved by the Ethics Committee of the Faculty of Medical Sciences, University of Kragujevac (Ref. No. 01-14321, 13 November 2017), entitled “Epidemiology of the most common health disorders”. These data are fully aggregated, without any identification data, and no patient approvals were required for the study.

## 3. Results

### 3.1. Search Results and the Studies’ Characteristics

The literature search yielded seventeen studies, of which fifteen were cohort studies and two were case–control studies [23,24,25,26,27,28,29,30,31,32,33,34,35,36,37,38,39]. There were six studies that estimated the incidence of diabetes mellitus type 2 after a SARS-CoV-2 infection, four studies that estimated the incidence of diabetes mellitus type 1, six studies that assessed both types, and one study that did not specify the assessed type (Table 1) [23,24,25,26,27,28,29,30,31,32,33,34,35,36,37,38,39]. The majority of the studies were conducted in the USA (10/16), with two of these ten studies also incorporating countries outside of the USA (including India, Malaysia, Bulgaria, Australia, Spain, and Taiwan), 6/16 studies were performed in Europe (Germany, England, Scotland, Spain, and Hungary), and one was performed in Israel. Most of the studies compared the incidence of new-onset diabetes mellitus in COVID-19 patients with healthy controls, while four studies included comparisons with persons who had upper respiratory tract infections, influenza, or non-COVID-19 pneumonia.

Detailed characteristics of the studies included in the review are presented in Table 2. The sample size of the studies ranged from 43 to 11,553,301. In most of the studies, the authors ascertained the presence of SARS-CoV-2 infection via electronic health records and data regarding the presence of corresponding ICD-10 codes for COVID-19, or a record of a positive PCR test, with only one study using data from serology testing for antibodies against SARS-CoV-2 spike proteins [29]. The diagnosis of new-onset diabetes mellitus type 1 and 2 was ascertained via the presence of corresponding ICD-10 codes in medical documentation or via a record of clinical diagnosis and records of increased HbA1c, fasting glucose, or dispensed glucose-lowering medication prescription. The follow-up period ranged from 24 h after the positive SARS-CoV-2 test to 365 days after the testing.

### 3.2. New-Onset Type 1 Diabetes Mellitus after SARS-CoV-2 Infection

There were four studies that reported the incidence of new-onset diabetes mellitus type 1 following SARS-CoV-2 infection. Three studies estimated the risk for new-onset diabetes mellitus type 1 compared to the general population, and the risk ranged from OR = 1.42 (95%CI = 1.38–1.46) [31] to OR = 3.74 (95%CI = 1.08–13.55) [29]. Compared to persons with non-COVID-19 respiratory infections, the risk for new-onset diabetes mellitus type 1 was HR = 1.96 (95%CI = 1.26–3.06) [32]. The risk was significantly increased both in males (from RR = 1.33 (95%CI = 1.17–1.50) [30] to RR = 1.49 (95%CI = 1.42–1.55) [31] and females (OR = 1.36; 95%CI = 1.30–1.42) [31]. One study assessed the risk by age and found that the risk for new-onset DM type 1 following COVID-19 was the highest at the age of 0–1 years (OR = 6.84 (95%CI = 2.75–17.02)) while at the age of 18–35, there was no difference in risk (OR = 0.97; 95%CI = 0.91–1.04) [31]. The same study investigated the differences in risk by ethnicity and found that the risk was the highest in American Indian/Alaskan Native (OR = 2.30; 95%CI = 1.86–2.82) and the lowest yet still significant risk increased in white persons (OR = 1.18; 95%CI = 1.13–1.23) [31]. 

### 3.3. New-Onset Type 2 Diabetes Mellitus after SARS-CoV-2 Infection

There were six studies that reported the incidence of new-onset diabetes mellitus type 2 following SARS-CoV-2 infection. Compared to the general population, the risk for new-onset diabetes mellitus type 2 ranged from HR = 1.46 (95%CI = 1.26–1.69) [27] to HR = 2.71 (95%CI = 2.45–2.99) [26]. Compared to persons with non-COVID-19 respiratory infections, the risk for new-onset diabetes mellitus type 2 in persons with COVID-19 ranged from HR = 1.30 (95%CI = 1.15–1.47) for persons with non-COVID pneumonia [26] to IRR = 1.51 (95%CI = 1.05–2.18) for persons with acute upper respiratory tract infection [25]. According to gender, the risk was significantly increased in males aged 65 and over [23]. The rates of DM type 2 following SARS-CoV-2 infection compared to the general population were 39.8 versus 7.5 per 1000 person-years for females and 48.5 versus 10.3 per 1000 person-years for males [26]. The risk for new-onset DM after SARS-CoV-2 infection was significantly increased in hospitalized patients (reported RD ranging from 2.21 (95%CI = 1.06–2.58) [24] to 3.71 (95%CI = 2.41–4.88) [23]. However, in SARS-CoV-2-positive persons who were not hospitalized with COVID-19, the risk was significantly lower in persons aged 18–65 (RD = 0.37, 95%CI = 0.24–0.58) [24], while in persons aged 65 and older who were not admitted to hospital for COVID-19, the risk difference for new-onset DM was not significant (RD = 0.94, 95%CI = 0.47–1.53) [23]. The rates of new-onset DM per 1000 person-years in persons with SARS-CoV-2 compared to the general population were increased the most in the black cohort (69.0 vs. 10.0), followed by Asian/Asian–British (58.8 vs. 14.4) and white (41.5 vs. 8.6) [26].

### 3.4. New-Onset Diabetes Mellitus after SARS-CoV-2 Infection

There were seven studies that assessed the risk of new-onset DM following SARS-CoV-2 infection but did not report data separately for type 1 or type 2 DM. The risk for new-onset DM ranged from HR = 1.19 (95%CI = 1.09–1.29) [37] to HR = 2.66 (95%CI = 1.98–3.56) [34]. According to sex, across the studies, the risk for new-onset DM was significantly increased in men (ranging from HR = 1.41 (95%CI = 1.37–1.45) [36] to OR = 2.56 (95%CI = 2.32–2.83) [35]. For females, the risk ranged from OR = 1.21 (95%CI = 0.88–1.68) [35] to RR = 1.5 (95%CI = 1.3–1.6) [33]. For the female sex, the study by Wander and coauthors [35] found that the risk was significantly increased only for women who were current smokers (OR = 1.43, 95%CI = 1.18–1.73). This study also reported that according to race, only black men had a significantly increased risk for new-onset DM. The patients who were hospitalized for COVID-19 had a significantly increased risk of new-onset DM, ranging from HR = 2.47 (95%CI = 1.86–3.29) [37] to HR = 2.73 (95%CI = 2.50–2.99) [36], and HR = 3.76 (95%CI = 3.24–4.37) for those admitted to the intensive care unit [36].

## 4. Discussion

This narrative review showed that the risk for new-onset DM type 1 following SARS-CoV-2 infection ranged from 1.42 to 3.74, compared to the control group. Studies that reported the incidence of new-onset DM type 2 following SARS-CoV-2 infection, compared to the control group, showed the risk for new-onset DM type 2 ranged from 1.30 to 2.71.

The research has shown that persons with pre-existing diabetes mellitus are at a greater risk of severe COVID-19 disease and mortality [40]. However, a meta-analysis has shown that persons with new-onset diabetes mellitus due to COVID-19 had the highest mortality rate (24.96%) compared to COVID-19 cases who had a history of diabetes mellitus (16.03%) and compared to non-diabetic COVID-19 cases (9.26%) [41]. COVID-19 patients with new-onset diabetes mellitus had the highest rate (45.85%) of adverse events (e.g., admission to the intensive care unit, intubation, severe COVID-19) compared to patients with a history of diabetes mellitus (20.69%) and non-diabetic patients with COVID-19 (15.29%). Therefore, identifying risk factors for the development of new-onset DM in COVID-19 patients is very important for preventing poor outcomes of COVID-19.

The risk of new-onset DM was increased in both genders. The results of a recent meta-analysis showed that the relative risk of new-onset DM following SARS-CoV-2 infection was increased by 2.08 in men and 2.15 in women [19]. Throughout the studies included in this review, the risk for new-onset DM was significantly increased for all races/ethnicities that were considered; however, the level of increase varied. The differences in the magnitude of risk increase could be at least partially explained by different risk trajectories in the past, in the time before the pandemic [31]. Furthermore, the risk for new-onset DM was higher in hospitalized patients than those not admitted to the hospital or intensive care unit. It is notable that when patients with COVID-19 develop new-onset DM it could have multiple and perplexing effects on the patient’s previous medical history and other health conditions [17].

It should be noted that a diagnosis of new-onset diabetes mellitus in COVID-19 patients could be a result of increased testing that occurs during COVID-19 diagnosis and treatment. Persons testing positive for SARS-CoV-2 could have had hyperglycemia, undiagnosed pre-diabetes, or undiagnosed diabetes and the increased access to healthcare services during COVID-19 diagnosis and therapeutic procedures could lead to a diagnosis of new-onset DM [42]. This could particularly be the case for some people who, due to various reasons, do not have access to healthcare services (e.g., poor people, people living in remote areas, etc.). A study in the UK observed the incidence of new-onset DM in a stratified manner over the follow-up period and showed that the risk for new-onset DM was increased significantly up to 4 weeks after SARS-CoV-2 diagnosis compared to the controls and at 5–12 weeks, but the difference became not significant at 13–52 weeks [38]. One of the possible explanations could be detection bias. Possible solutions for addressing detection bias could include increased access to healthcare services, particularly screening for diabetes, but also the application of various statistical techniques [43] in studies aimed at assessing the new onset of diabetes in COVID-19 patients. Future research should strive to incorporate longer follow-ups and investigate the incidence of new-onset diabetes mellitus at various time points throughout that follow-up period.

Furthermore, it should be noted that the reported estimates of the risk of new-onset diabetes mellitus in patients with COVID-19 should be regarded with caution due to possible overestimation. Namely, during the course of treatment for COVID-19, the patients can receive glucocorticoids and experience hyperglycemia that can unmask diabetes that was not recognized until then [24]. Research shows that about one-third of patients treated with glucocorticoids develop hyperglycemia [44]. According to the results of a meta-analysis, about one in four COVID-19 patients develop COVID-19-associated hyperglycemia [41]. However, the studies included in this review did not provide detailed data on the use of glucocorticoids during the hospitalization for COVID-19, dosage, or duration of their use. Still, two studies reported performing a sensitivity analysis [38] and additional adjustments [36] to account for the use of steroids, with Horberg et al. [38] reporting that the use of corticosteroids likely had no association with the increased risk of diabetes mellitus noted in patients with COVID-19. Future studies should provide more details on the use of glucocorticoids in the treatment of COVID-19 in order to assess its true impact on the incidence of new-onset DM following COVID-19 diagnosis. The question remains as to whether the risk of developing DM in COVID-19 patients is underestimated as a consequence of the presence of undiagnosed or asymptomatic SARS-CoV-2 infections in the control groups. Additionally, due to the different availability of testing and the different frequency of COVID-19, the results of some studies that were carried out during different periods of the pandemic should be interpreted with caution.

A precise explanation of diabetes occurrence in COVID-19 patients remains unclear. It is important to point out that this relationship is supported by a previous study that investigated the pathogenesis of pancreatic lesions and glucose intolerance in SARS patients during the SARS coronavirus (SARS-CoV) global alert in March 2003, which revealed that angiotensin-converting enzyme 2 (ACE2) expression in the exocrine and endocrine tissues of the pancreas suggested that SARS-CoV may damage islets and cause acute insulin-dependent diabetes mellitus [45]. In recent years, increasing evidence demonstrated that ACE2 is expressed in insulin-producing β-cells, pancreas microvasculature pericytes, and ductal cells, indicating a potential link between SARS-CoV-2 and diabetes through either the infection of pancreatic microvasculature or ductal cells, or by direct β-cell virus tropism [46,47].

The WHO and the United Nations Sustainable Development Goals (UNSDGs) aim for a one-third premature mortality reduction for non-communicable diseases through prevention and treatment; they also aim to promote mental health and well-being by 2030 [48]. Along with the effects of the COVID-19 pandemic, due to aging population, insufficient knowledge of the etiology leads to very limited possibilities of prevention, management, and the increase in the prevalence of certain risk exposures (i.e., overweight/obesity, physical inactivity, dietary risk, alcohol use, etc.), it would be difficult to expect that the UNSDG’s goal of reducing mortality from non-communicable diseases (diabetes) by one-third by 2030 can be achieved. Following the onset of the COVID-19 pandemic, numerous studies demonstrated a rise in both all-cause and COVID-19-related mortality among patients with diabetes, which is accompanied by concern about a possible bi-directional relationship between these two diseases [1,2,49,50]. Further efforts to identify risk factors and pathogenesis of COVID-19 and diabetes mellitus could help improve strategies for implementing more effective prevention measures and for better management of the potential long-term consequences of these two diseases.

### Limitations of the Study

This research had several sources of limitations. Primarily, due to the heterogeneity of the research studies conducted so far regarding diabetes mellitus following SARS-CoV-2 infection, the results are not conclusive or uniform. Further, there were inconsistencies across studies with respect to how diabetes mellitus and COVID-19 cases were determined. Most studies that examined the incidence of diabetes mellitus reported various follow-up periods since SARS-CoV-2 occurred. Namely, some research used a retrospective study design (with well-known shortcomings), with important differences in the size of the study sample, with a very uneven selection of variables that could be of importance for the diabetes mellitus following SARS-CoV-2 infections. Additionally, most studies did not estimate the level of glycemia prior to COVID-19, which could have helped in elucidating the effect of SARS-CoV-2 infection on developing diabetes mellitus. Additionally, the issue of information bias and response bias always exists, especially in the conditions of the ongoing pandemic. These sources of bias can be reduced by careful planning of study design, data collection, and the use of standardized laboratory testing and validated definitions of exposure and outcome. Finally, some studies assessed the risk for new-onset diabetes mellitus by looking at both type 1 and type 2 together, without presenting data for each type separately. Additionally, studies were performed at various time points throughout the pandemic with different circumstances regarding living conditions and access to healthcare. Hence, further research is necessary to better clarify the link between COVID-19 and new-onset DM type 1 and type 2.

## 5. Conclusions

This narrative review indicated that SARS-CoV-2 infection carries a significant risk for diabetes mellitus occurrence. Our paper showed that the risk for new-onset DM type 1 following SARS-CoV-2 infection ranged from 1.42 to 3.74 and the risk for new-onset DM type 2 ranged from 1.30 to 2.71. The most commonly identified risk factors associated with diabetes mellitus following SARS-CoV-2 infection were severity of disease, age, ethnicity, ventilation, and smoking habits. Further studies are necessary to determine the burden of diabetes mellitus after SARS-CoV-2 infection and its predictors in order to plan and create effective measures for successful diabetes mellitus management.

## Figures and Tables

**Table 1:** Diabetes mellitus (DM) after SARS-CoV-2 infection: a summary of the studies.

Author, Year [Ref. No.]	Location	Study Design	Study Population	DM Type	Risk Estimate (95% Confidence Interval)
Cohen, 2021 [23]	USA	Retrospective cohort	Persons ≥ 65 enrolled in a Medicare Advantage plan; comparison groups—2020 group, historical 2019 group, historical group with viral LRTI	2	HR = 1.96 (1.6–2.4) *p* < 0.001
Daugherty, 2021 [24]	USA	Retrospective cohort	Persons 18–65 with a continuous enrolment in the health plan; comparison groups—2020 group, historical 2019 group, historical group with viral LRTI	2	HR = 1.83 (1.60–2.10), *p* < 0.001
Rathmann, 2022 [25]	Germany	Retrospective cohort	Patients from the database of a representative panel of physicians’ practices with either mild COVID-19 or acute upper respiratory tract infection	2	IRR = 1.51 (1.05–2.18)
Tazare, 2022 [26]	England	Retrospective cohort	Patients ≥ 18 years, registered with a general practice, with COVID-19, or non-COVID-19 pneumonia and general population	2	For COVID-19 vs. general population HR = 2.71 (2.45–2.99), for COVID-19 versus non-COVID pneumonia HR = 1.30 (1.15–1.47)—fully adjusted models
Birabaharan, 2022 [27]	USA, India, Malaysia, Bulgaria, Australia, Spain, Taiwan	Retrospective cohort	Patients with mild, moderate, and severe COVID-19 and patients with influenza	2	HR = 1.46 (1.26–1.69)
Hernandez-Romieu, 2022 [28]	USA	Retrospective cohort	PCORnet system	2	Nonhospitalized adults: PR = 0.9 (0.85–0.96), Nonhospitalized children/young adults PR = 1.27 (0.75–2.14)
Herczeg, 2022 [29]	Hungary	Case–control study	Children 0–18 years hospitalized with new-onset T1DM and known T1DM children as the controls (unvaccinated, otherwise healthy)	1	OR = 3.74 (1.08–13.55), *p* = 0.04
McKeigue, 2022 [30]	Scotland	Matched case–control	The REACT-SCOT (Rapid Epidemiological Analysis of Comorbidities and Treatments as Risk Factors for COVID-19 in Scotland)	1	RR = 2.62 (1.81–3.78), *p* = 3 × 10^−7^
Qaedan, 2022 [31]	USA	Retrospective cohort	Cerner Real-World Data—electronic health records	1	OR = 1.42 (1.38–1.46), *p* < 0.001
Kendal, 2022 [32]	50 US states + 14 countries	Retrospective cohort	TriNetX Analytcs Platform database—patients 18 years old or younger with a SARS-CoV-2 infection or with a non-SARS-CoV-2 respiratory infection	1	HR = 1.96 (1.26–3.06)
Ayoubkhani, 2021 [33]	England	Retrospective cohort	NHS England data on individuals with a hospital episode due to COVID-19 and general population control without COVID-19	Both	28.7 (26.0–31.7) per 1000 person-years in COVID-19 patients vs. 8.2 (39.8–45.5) per 1000 person-years in the control group
Barrett, 2022 [34]	USA	Retrospective cohort	Persons < 18 years from the IQVIA and HealthVerity databases	Both	IQVIA: HR = 2.66 (1.98–3.56) HealthVerity HR = 1.31 (1.20–1.44)
Wander, 2022 [35]	USA	Retrospective cohort	Veterans Health Administration data	Both	0.46% among COVID-19 patients vs. 0.19% among non-COVID-19 patients at 120 days; 0.60% among COVID-19 patients vs. 0.32% among non-COVID-19 patients
Xie, 2022 [36]	USA	Retrospective cohort	US Department of Veterans Affairs	Both	HR = 1.40 (1.36–1.44)
Reges, 2023 [37]	Israel	Retrospective cohort	All Clait members ≥ 25 years	Both	HR = 1.19 (1.09–1.29)
Rezel-Potts, 2022 [38]	UK	Matched cohort	CPRD Aurum	Both	Up to 4 weeks post-COVID-19 RR = 1.81 (1.51–2.19), *p* < 0.001
Horberg, 2022 [39]	USA	Retrospective cohort	Kaiser Permanente health system	Not specified	30–120 days post-COVID-19 RR = 1.20 (1.03–1.38)

Abbreviations: HR—hazard ratio; IRR—incidence rate Ratio; PR—prevalence ratio; OR—odds ratio; RR—risk ratio.

**Table 2:** Diabetes mellitus (DM) after SARS-CoV-2 infection: characteristics of the studies.

Author, Year [Ref. No.]	Sample Size	SARS-CoV-2 Diagnosis Ascertainment	DM Diagnosis	Time between SARS-CoV-2 Infection and DM Diagnosis	Stratified Risk Estimates (95% Confidence Interval)
Cohen, 2021 [23]	SARS-CoV-2 infected 133,366, 3 control groups, 2020 comparison group (87,337), historical 2019 comparison group (88,070), historical comparison group with LRTI (73,490)	Administrative claim with an ICD-10 code, PCR test, or clinical diagnosis	Administrative claims data with ICD-10 codes	Post-acute phase (≥21 days after the index date)	2019 RD = 1.72 (1.41–2.1) *p* < 0.001; LRTI RD = 1.23 (1.02–1.48), *p* = 0.005 By age: 65–74 RD = 1.34 (0.77–1.94), *p* < 0.001, 74+ RD = 1.71 (1.09–2.26), *p* < 0.001 By race: white RD = 1.36 (0.87–1.84), *p* < 0.001, black RD = 3.19 (0.94–5.48), *p* < 0.001 By gender: female RD 1.21 = (0.62–1.73), *p* < 0.001, male: RD = 1.97 (1.19–2.86), *p* < 0.001 By COVID-19 hospitalization: not hospitalized RD = 0.94 (0.47–1.53), *p* < 0.001, hospitalized RD = 3.71 (2.41–4.88), *p* < 0.001
Daugherty, 2021 [24]	SARS-CoV-2 infected 193,113	Administrative claim with an ICD-10 code, PCR test, or clinical diagnosis	Administrative claims data with ICD-10 codes	Post-acute phase (≥21 days after the index date)	RD = 0.47 (0.35–0.59), *p* < 0.001 By age: 18–34 RD = 0.2 (0.05–0.32), *p* < 0.001, 35–50 RD = 0.51 (0.17–0.75), *p* < 0.001, 50+ RD = 0.74 (0.39–1.3), *p* < 0.001 By sex: female RD = 0.48 (0.32–0.75), *p* < 0.001, male RD = 0.47 (0.1–0.55), *p* < 0.001 By COVID-19 hospitalization: not hospitalized RD = 0.37 (0.24–0.58), *p* < 0.0001, hospitalized RD = 2.21 (1.06–2.58), *p* < 0.001
Rathmann, 2022 [25]	SARS-CoV-2 infected 35,865, acute upper respiratory tract infection 35,865	ICD-10 codes data	ICD-10 codes data	1–365 days after the index date	/
Tazare, 2022 [26]	77,347 patients discharged after COVID-19 hospitalization, 127,987 patients discharged with non-COVID pneumonia, 386,669 general population	Presence of a diagnostic code either in a general practice record, hospital, or on a death certificate	Presence of a diagnostic code either in a general practice record, hospital, or on a death certificate	The entire follow-up, 0–29 days, 30–59 days, 60–89 days, 90–120 days, 120+ days	Age-sex adjusted: COVID-19 vs. general population HR = 4.20 (3.86–4.57), COVID-19 vs. non-COVID-19 pneumonia HR = 1.46 (1.31–1.63) Rates per 1,000 person-years of T2DM after COVID-19 compared to the general population: By age: 18–49 33.2 vs. 3.1, 50–59 51.8 vs. 7.2, 60–69 59.2 vs. 10.0, 70–79 47.5 vs. 12.0, 80+ 38.4 vs. 12.6 By sex: female 39.8 vs. 7.5, male 48.5 vs. 10.3, By ethnicity: white 41.5 vs. 8.6, mixed 13.5 vs. 8.6, Asian/Asian-British 58.8 vs. 14.4, black 69.0 vs. 10.0
Birabaharan 2022, [27]	10,436 patients with moderate/severe COVID-19 and 10,951 persons with moderate/severe influenza, 35,865 persons with mild COVID-19	ICD-10 codes data or PCR	ICD-10 codes data	1–180 days after the index event	/
Hernandez-Romieu, 2022 [28]	144,768 nonhospitalized and 23,933 hospitalized COVID-19-positive, 1,227,510 nonhospitalized and 394,675 hospitalized with a negative COVID-19 test	PCR or antigen testing	ICD-10 codes	31–150 days after COVID-19 testing	Hospitalized adults: PR = 2.03 (1.87–2.19) Hospitalized children/young adults PR = 2.14 (1.13–4.06) Mechanically ventilated adults: PR = 2.25 (1.82–2.77)
Herczeg, 2022 [29]	21 newly-diagnosed children T1DM (11 SARS-CoV-2 seropositive) and 22 with pre-existing disease (5 SARS-CoV-2 seropositive)	Serology testing for antibodies against SARS-CoV-2 spike proteins at admission with new-onset T1DM or within three months after discharge	Clinical diagnosis	/	/
McKeigue, 2022 [30]	11,552,227 SARS-CoV-2 positive without diabetes and 1074 SARS-CoV-2-positive with incident diabetes	PCR test	Clinician’s record of diagnosis in the SCI, Diabetes registry	0–30 days	By sex: male RR = 1.33 (1.17–1.50), *p* < 5 × 10^−6^
Qaedan, 2022 [31]	2,489,266 patients with COVID-19 (of whom 5163 had TD1M), 24,803,603 non-COVID-19 patients (of whom 36,348 had TD1M)	Confirmed diagnosis code or swab result	ICD-10 code at least 24 h after the date of confirmed COVID-19 diagnosis	/	By age: 0–1 OR = 6.84 (2.75–17.02), 2–5 OR = 2.19 (1.68–2.85), 6–12 OR =2.04 (1.78–2.33), 13–17 OR = 1.56 (1.38–1.76), 18–35 OR = 0.97 (0.91–1.04), 36–50 OR = 1.54 (1.44–1.64), 51–65 OR = 1.77 (0.66–1.88), >65 OR = 1.43 (1.34–1.52) By sex: female OR = 1.36 (1.30–1.42), male OR = 1.49 (1.42–1.55) By ethnicity: American Indian/Alaskan Native OR = 2.30 (1.86–2.82), Asian/Pacific Islander OR = 2.01 (1.61–2.53), black OR = 1.59 (1.47–1.71), Hispanic OR = 1.52 (1.41–1.63), white OR = 1.18 (1.13–1.23) By marital status: married OR = 1.34 (1.27–1.42), not-married OR = 1.35 (1.30–1.39)
Kendall, 2022 [32]	285,628 patients with COVID-19, 285,628 patients with non-COVID-19 respiratory infections	Lab test or ICD-10 code	ICD-10 code	1, 3, and 6 months after infection	3 months: HR = 2.10 (1.48–3.00) 6 months: HR = 1.83 (1.36–2.44)
Ayoubkhani, 2021 [33]	47,780 patients with COVID-19, 47,780 matched control group	ICD-10 codes—a positive laboratory test or a clinical diagnosis	ICD-10 codes from primary care and hospital records	After discharge from the hospital	By sex: male RR = 1.5 (1.4–1.6), female RR = 1.5 (1.3–1.6) By age group: <70 years RR = 1.7 (1.6–1.8), = > 70 years RR = 1.3 (1.2–1.4) By ethnic group: white RR = 1.4 (1.3–1.5), non-white RR = 1.5 (1.3–1.7)
Barrett, 2022 [34]	IQVIA: 80,893 COVID-19 patients and 404,465 non-COVID-19 patients, 404,465 ARI and 808,930 non-ARI patients HealthVerity 439,439 COVID-19 patients and 439,439 non-COVID-19 patients	ICD-10 codes or a positive PCR test	ICD-10 codes claims > 30 days after the index date	>30 days after the index date	Diabetes incidence per 100,000 person-years, COVID-19 vs. non-COVID-19 IQVIA: Overall 316 vs. 118, by age 0–11 years 261 vs. 76, 12–17 years 346 vs. 142, by sex female 313 vs. 123, male 318 vs. 114 HealthVerity Overall 399 vs. 304, by age 0–11 years 211 vs. 118, 12–17 526 vs. 381, by sex female 427 vs. 339, male 370 vs. 268
Wander, 2022 [35]	SARS-CoV-2-positive, 126,710, no positive swabs, 2,651,058	Positive nasal swabs	ICD-10 codes data	At 120 days after the index date and during the entire follow-up period	At 120 days: By sex: men OR = 2.56 (2.32–2.83, women OR = 1.21 (0.88–1.68) By ethnicity and sex: white men OR = 1.09 (0.85–1.40), white women OR = 1.31 (0.75–2.29), black men OR = 1.61 (1.25–2.09), black women OR = 1.44 (0.85–2.55), latinx men OR = 1.49 (1.31–1.70), latinx women OR = 1.06 (0.77–1.48) By smoking and sex: former smoker men OR = 1.20 (1.11–1.31), former smoker women OR = 1.06 (0.86–1.32), current smoker men OR = 1.61 (1.49–1.75), current smoker women OR = 1.43 (1.18–1.73)
Xie, 2022 [36]	181,280 COVID-19 persons, 4,118,441 contemporary controls, 4,286,911 historical controls	COVID-19-positive test	ICD-10 codes or an HbA1c measurement of >6.4%	>30 days after the index date	By age: ≤65 years HR = 1.36 (1.31–1.41), >65 years HR = 1.43 (1.37–1.49) By race: white HR = 1.37 (1.37–1.42), black HR = 1.41 (1.37–1.45) By sex: male HR = 1.41 (1.37–1.45), female HR = 1.33 (1.21–1.45) By care setting: nonhospitalized HR = 1.25 (1.21–1.29), hospitalized HR = 2.73 (2.50–2.99), intensive care HR = 3.76 (3.24–4.37)
Reges, 2023 [37]	157,936 patients with COVID-19 and 157,936 controls	PCR test	ICD-10 code, HbA1c ≥ 6.5%, two documentations of fasting glucose ≥ 126 mg/dL, dispensed prescription of glucose-lowering medication	Baseline, 1, 2, 3, and 4 months after COVID-19 diagnosis	Nonhospitalized: HR = 1.08 (0.99–1.18) Hospitalized: HR = 2.47 (1.86–3.29) Hospitalized with severe disease: HR = 3.33 (1.94–5.72)
Rezel-Potts, 2022 [38]	431,193 COVID-19-positive patients,	Clinical or laboratory confirmation, PCR test		Up to 52 weeks post-COVID-19 diagnosis	5–12 weeks post-COVID-19 diagnosis RR = 1.27 (1.11–1.46), *p* < 0.001 13–52 weeks post-COVID-19 diagnosis RR = 1.07 (0.99–1.16), *p* = 0.07
Horberg, 2022 [39]	31,390 COVID-19-positive patients (28,118 for the matched cohort), control 70,293 persons	PCR test result	ICD-10 codes	0–30 days post-COVID-19 test, 30–120 days post-COVID-19 test	0–30 days post-COVID-19 test RR = 1.96 (1.41–2.74)

Abbreviations: LRTI—lower respiratory tract infection; RD—risk difference, HR—hazard ratio; PR—prevalence ratio; T1DM—type 1 diabetes mellitus; OR—odds ratio; RR—risk ratio.

## Data Availability

Data are contained within the article.
